# Low activity of lytic pelagiphages in coastal marine waters

**DOI:** 10.1038/s41396-018-0185-y

**Published:** 2018-06-05

**Authors:** Laura Alonso-Sáez, Xosé Anxelu G. Morán, Martha RJ Clokie

**Affiliations:** 1 AZTI, Marine Research Division, Sukarrieta, Spain; 2 Centro Oceanográfico de Gijón/Xixón, IEO, Gijón, Xixón, Spain; 3 King Abdullah University of Science and Technology (KAUST), Red Sea Research Center, Biological and Environmental Sciences and Engineering Division, Thuwal, Saudi Arabia; 4 Department of Infection, Immunity and Inflammation, University of Leicester, Leicester, UK

## Abstract

Phages infect marine bacteria impacting their dynamics, diversity and physiology, but little is known about specific phage–host interactions in situ. We analyzed the joint dynamics in the abundance of phage-related transcripts, as an indicator of viral lytic activity, and their potential hosts using a metatranscriptomic dataset obtained over 2 years in coastal temperate waters of the NE Atlantic. Substantial temporal variability was identified in the expression levels of different phages, likely in response to host availability. Indeed, a significant positive relationship between the abundance of transcripts from some of the most abundant phage types (infecting SAR11, SAR116 and cyanobacteria) and their putative hosts was found. Yet, the ratio of increase in phage transcripts per host cell was significantly lower for pelagiphages than for the HMO-2011 phage, which infects SAR116. Despite the high abundance of pelagiphages in the ocean, they may be less active than other phage types in coastal waters.

The study of phage–host microbial interactions in the ocean has been challenging due to the difficulty of isolating environmentally important microbes and their phages, as well as of assessing the true diversity of viral infections in nature. New approaches such as metagenomics [1, 2] and single-cell and single-virus genomics [3, 4] have recently targeted the genomic repertoire of viruses thriving as free particles or replicating in uncultivated microbes. Yet, key questions remain unanswered, such as which bacterial phylotypes are most susceptible to viral predation, and how phage–host interactions change across environmental gradients.

To determine how active viral infection impacts seasonal bacterial dynamics, we identified transcripts of phage origin in eight metatranscriptomes collected over 2 years from a coastal station in the S Bay of Biscay (E2 Gijón/Xixón) and compared the data to the abundance of putative bacterial hosts. From 4.2 million mRNA transcripts, 7616 phage-origin hits were identified by a combined BLASTx and BLASTn query to the NCBI Refseq database (see Supplementary Information). Additionally, more than 2600 significant hits to phage genomes recently sequenced by cultivation-independent methods were identified based on nucleotide homology (Supplementary Table S1). Some of the most abundant marine phage types discovered to date abounded in our dataset, including phages infecting cyanobacteria (cyanophages), SAR11 (pelagiphages) and the SAR116 clade (HMO-2011 phage, [5], Fig. 1). The transcript abundance of these three phage types was substantially higher when identified by protein similarity (BLASTx search) compared to nucleotide similarity (BLASTn search), even when the Refseq nucleotide database was augmented with recently sequenced phages (Supplementary Figure S1). Our results thus indicate a substantial variation at the nucleotide level within these phage types, in line with recent results of widespread marine phages by single-virus genomics [3]. The phage vSAG-37-F6, discovered in the latter study, recruited the highest number of hits by nucleotide similarity (Supplementary Figure S2). Thus, we provide evidence of high in situ activity of what is likely one of the most abundant marine virus species [3]. In general, substantial temporal variability was identified in the expression levels of different phages across the seasonal gradient (Fig. 1), presumably in response to host availability.

**Fig. 1:**
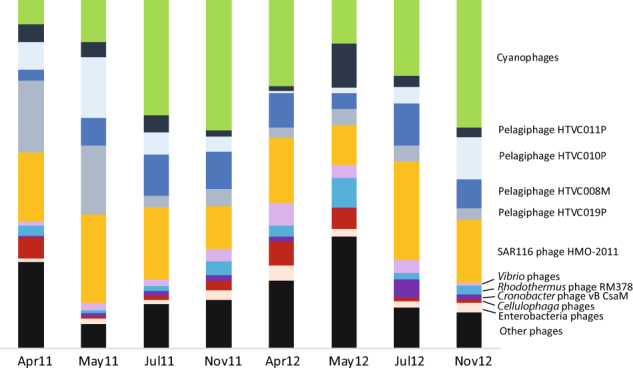
Temporal dynamics in the abundance of transcripts of phages targeting different bacterial clades. Phage transcripts have been identified based on protein similarity (BLASTx search) against the Refseq database and normalized by the size of phage genomes and metatranscriptomic libraries (see Supplementary Text). Only those phage types contributing >2% of total phage transcripts are shown

Putative hosts have been identified for some of the phages found in our dataset according to their isolation source or genomic information (Supplementary Table S1). Based on the similarity of metatranscriptomic reads to phages with known hosts and assuming phage–host specificity, the relationships between the abundance of transcripts of pelagiphages, cyanophages and HMO-2011 and their respective putative host cells were explored to test whether host density promoted the viral lytic response [6]. Significant correlations (Spearman test, *P* ≤ 0.05, *n* = 8) were found for the three groups (Fig. 2), indicating that phage–host relationships could be detected by this approach in situ, even at a broad phylogenetic level. These relationships were significant when phage transcript abundance was estimated based on protein similarity. However, they were not significant in the case of pelagiphages and cyanophages when estimated based on nucleotide similarity (Spearman test, *P* > 0.05, Supplementary Figure S1). This again suggests a substantial diversity within these phages only detectable at the ‘catch all’ protein level. Other abundant viruses were likely infecting members of Flavobacteria, Verrucomicrobia, SAR86 and SAR92 clades (Supplementary Figure S2). Yet, for the latter groups, only the abundance of transcripts of phage type AAA160-D02 and their putative host, the SAR92 clade, were positively correlated (Spearman *R* = 0.78, *P* = 0.02).

**Fig. 2:**
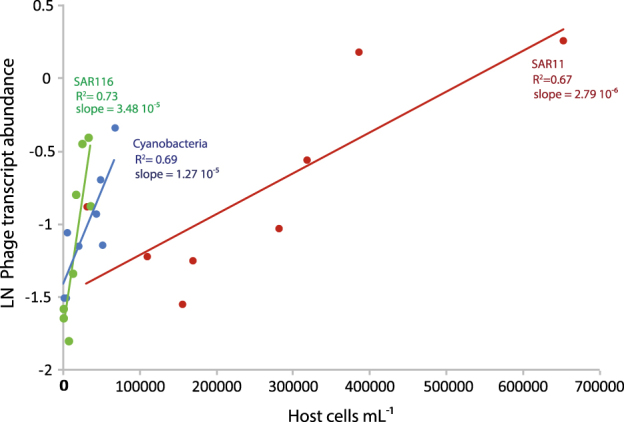
Relationship between the natural log-transformed abundance of phage transcripts in the metatranscriptomes and the abundance of their respective host cells for the HMO-2011 phage (targeting SAR116, in green), cyanophages (in blue) and pelagiphages (in red). Phage transcripts were identified based on protein similarity (BLASTx search) against the Refseq database and their abundance was normalized by the size of phage genomes and metatranscriptomic libraries. The coefficients of determination (*R*^2^) and slopes of the regression lines are shown

Interestingly, the slopes of the phage–host abundance regressions were significantly different for HMO-2011 and pelagiphages (ANCOVA test, *P* < 0.01), and marginally significant between HMO-2011 and cyanophages (ANCOVA test, *P* = 0.051), or cyanophages and pelagiphages (ANCOVA test, *P* = 0.058). Taking phage transcript abundance as an indicator of viral lytic activity, likely related with burst size and lysis rates, our results indicate a higher activity of HMO-2011 as compared to pelagiphages in situ. In support of our results, in the experiments performed in the laboratory, the latent periods of HMO-2011 are substantially shorter than pelagiphages (6 h versus 16–22 h) and burst sizes are one order of magnitude higher (500 versus 9-49, [5, 7]), which is consistent with faster infection cycles.

The lower ratio of pelagiphage transcripts per host cell (ca. one order of magnitude lower compared with cyanophages or HMO-2011) could be explained by the presence of a lower number of infected cells, possibly related to phage protection mechanisms transmitted by homoimmunity or recombination in SAR11 populations [7]. It has also been suggested that viruses cannot invade high-density host populations due to low rates of host growth and viral lysis [8]. SAR11 comprises some of the most abundant bacterial populations in the ocean [9]. While their in situ metabolic activity shows great variability [10, 11], their growth rates are typically low both in cultures [12] and in natural samples [13, 14], including our study site [15]. Thus, the lower phage–host abundance regression slope found for pelagiphages could be related to a slower growth of SAR11 cells, which also show weak transcriptional responses [16]. Yet, an occasional increase in the growth rate of SAR11 (above 1 day^−1^), concomitant with a replacement of the dominant SAR11 taxon in situ took place at our study site in May 2012 [15]. The composition of active pelagiphages also showed marked changes in this sample, with maximum abundance of HTVC011P transcripts (Fig. 1). These results would be consistent with the existence of several SAR11 strains characterized by different life strategies and phage susceptibility [17].

In summary, our study highlights the importance of studying the phage–host dynamics in situ, providing evidence of a seasonality in these interactions, and show different activity of phages targeting environmentally relevant microbial clades. The discovery of large numbers of pelagiphages in the ocean has suggested that the importance of viral control upon SAR11 populations could be higher than previously assumed [7]. However, the relatively low abundance of pelagiphage transcripts at high host densities found here may indeed suggest that a pseudolysogenic or chronic infection state may be more prevalent than the lytic cycle. The high activity of phages targeting SAR116 and cyanobacteria would be consistent with the large representation of the latter groups in transcriptionally active communities [18, 19] despite their relatively lower in situ abundance [20]. Further research combining measurements of viral and host replication rates will be key to elucidate the degree of viral control upon major microbial players of ocean biogeochemistry.

## Electronic supplementary material

Supplementary Information
