# Supplementary material for: Low activity of lytic pelagiphages in coastal marine waters
Source: ISME J. 2018 Jun 5;12(8):2100–2. doi: 10.1038/s41396-018-0185-y (PMC6052063; doi:10.1038/s41396-018-0185-y)
Supplement: Supplementary file 1 — Supplementary Information [file 41396_2018_185_MOESM1_ESM.pdf]

Supplementary information (ISME J Short Communication)

Low activity of lytic pelagiphages in marine coastal waters

Alonso-Sáez L, Morán XAG, Clokie MRJ

Methods

Samples collection. Eight metatranscriptomic samples were collected at a coastal station in the Southern Bay of Biscay (station Gijón-Xixón E2, 43.67ºN, 5.58ºW), where a long-term monitoring of basic parameters (Morán et al. 2015) and a multiyear study of bacterial community dynamics (Alonso-Sáez et al. 2015) are available. Metatranscriptomic samples were collected in spring (April and May), summer (July) and autumn (November) along two years (2011 and 2012). As in 2012 we could not collect samples in April due to weather conditions, and two samples were collected in May (2nd and 23rd May). The early May sample (2nd May) has been designated as "April 2012", for convenience. Samples (from 4.5 to 11 L) were collected 1-3 hours after midday from a depth of 5m and immediately filtered using 3-µm pore-size polycarbonate pre-filters and 0.22-µm pore-size polycarbonate filters (Millipore) to retain the bacterial fraction. The 0.22-µm filters were placed in Whirl-Pak bags containing 2 mL of RLT buffer (Qiagen) with 10 µL of beta-mercaptoethanol, flash frozen in liquid nitrogen and stored at -80ºC until analysis. Total time from sample collection to flash freezing of the filters ranged between 15 and 20 minutes.

RNA processing. RNA was extracted following a protocol previously detailed (Poretsky et al., 2009; Gifford et al., 2013). Briefly, filters were shattered with a mallet, vortexed in falcon tubes containing Power Soil beads (Mobio), and the lysate was mixed with 70% ethanol (1:1 volume). The RNA extraction was carried out with the RNeasy Mini Kit (Qiagen). RNA was treated with TurboDNAse (Ambion) and Ribosomal RNA (rRNA) was depleted using the mRNA-only isolation kit (Epicentre) and the MicrobeExpress and MicrobeEnrich kits (Ambion). The enriched mRNAs were linearly amplified using the Message Amp II- Bacteria kit (Ambion), reverse transcribed to double-stranded complementary DNA (cDNA) with the Universal Riboclone cDNA synthesis system (Promega) and purified with the QIAQuick PCR purification kit (Qiagen). The cDNA samples were sequenced in an Illumina Miseq platform. Raw sequences from the metatranscriptomic libraries used in this study have been deposited in the European Nucleotide Archive (ENA; www.ebi.ac.uk/ena) under the following accession numbers ERS1836494- ERS1836501.

Bioinformatic analysis. After an initial quality trimming of the reads, ribosomal RNAs were identified using a SILVA reference database and removed from the database. The sequence

of phiX174, used as control in Illumina platforms, was also deleted from the database. Sequences of phage origin were identified based on the homology to microbial genomes in the National Center for Biotechnology Information's (NCBI; http://www.ncbi.nlm.nih.gov) Refseq database using a two-step procedure. First, we carried out a BLASTx analysis (bitscore cutoff ≥ 40) against the Refseq protein database (version 63, January 2014), which contains protein sequences from representative genomes of bacteria, eukaryotes and viruses. Taxonomic affiliation of the reads was assigned based on the top-score RefSeq hit. Sequences with top-hits assigned to phage proteins were identified using a text-based query with the word 'phage', and visual inspection of the annotations to confirm matches to phage genomes. The phage-origin reads retrieved (13679 sequences) were subsequently compared against the NCBI Refseq genomic database (downloaded on 26 March 2014) by BLASTn (bitscore cutoff ≥ 50) to confirm their assignment to viral genomes at the nucleotide level. Those reads that had a non-viral genome as top-hit in this second BLAST search were excluded from subsequent analysis. This was mainly the case of photosynthetic genes in cyanophages, which share a high similarity in the phage and host cyanobacterial genomes and, thus, at our level of resolution, could not be reliably assigned taxonomically at the protein level. In the final dataset obtained from the search against the Refseq protein database we kept only those reads that had a phage genome as top-hit both at the amino acid and nucleotide levels, or reads that were highly similar to phage proteins (i.e., with a BLASTx top-hit to a phage genome) but no significant similarity to any genome in the Refseq nucleotide database (7616 sequences in total). The abundance of phage transcripts was normalized by the size of phage genomes and metatranscriptomic libraries (i.e. the number of significant BLASTx hits were divided by the phage genome size in Mbp, multiplied by 100 and divided by the total number of mRNA reads in each metatranscriptome).

In order to check for potential cross-recruitment of BLAST hits among different phages, we compared the taxonomic affiliation of the BLASTx and BLASTn top-hits in those cases where significant hits had been detected in both searches. We found that the cyanophage origin of the transcripts was highly consistent (BLASTx results confirmed in 93% cases by BLASTn), but the assignment of transcripts to specific *Synechococcus* and *Prochlorococcus* phage genomes were often inconsistent due to the high similarity at the amino acid level of some of their genes. Therefore, their abundance has been pooled as "cyanophages" without further differentiation between both genera. For the other phages, the taxonomic assignment of BLASTx hits was confirmed at the nucleotide level in 100% of cases for the HMO-2011 phage

and in 96% of cases for pelagiphages (100% of cases for HTVC019P and HTVC010P, 97% of cases for HTVC011P and in 61% of cases for HTVC008M).

Additionally, in order to identify in the metatranscriptomes newly sequenced phages whose genomes became available after the initial BLAST search against the Refseq databases, a custom genomic database was built including viral genome sequences available from single-cell amplified genomes (Labonté et al. 2015), single-virus amplified genomes (Martinez-Hernandez et al. 2017) and viral fosmids from surface (Mizuno et al. 2013) and deep oceanic waters (Mizuno et al. 2016). The genomes of non-cyanobacterial phages from the NCBI Refseq genomic database with significant hits in the metatranscriptomes (after the initial BLAST search) were also included in this custom database. Cyanophages (from Refseq or the other sources) were excluded from the database due to the high potential of recruiting non-viral cyanobacterial hits by BLAST search, because of the high similarity of some of their genes with those of cyanobacterial origin (as explained above).

Determination of bacterial hosts abundance. Different approaches were used in order to obtain the most accurate available estimates of the bacterial host *in situ* abundance. In the case of cyanobacteria, total abundance of *Synechococcus* and *Prochlorococcus* was determined by flow cytometry as explained in Calvo-Díaz and Morán (2006). Estimates of *in situ* cell abundance of SAR11 were obtained using CARD-FISH with the probe SAR11-441R (Morris et al. 2002) as previously described (Arandia-Gorostidi et al. 2017). Finally, in order to estimate the cell abundance of SAR116 cells, as no CARD-FISH data were available, we multiplied the relative abundance of this taxon in the samples obtained by 16S rRNA amplicon sequencing, to the total bacterial counts estimated by flow cytometry (following Alonso-Sáez *et al.*, 2015). Despite the existence of potential biases in the latter quantification associated with the sequencing procedure, we have shown that this strategy produced a reliable representation of the abundance of different populations in our dataset (see results in Alonso-Sáez *et al.,* 2015).

References

Arandia-Gorostidi N, Huete-Stauffer TM, Alonso-Sáez L, Morán XAG. (2017) Testing the Metabolic Theory of Ecology with marine bacteria: Different temperature sensitivity of major phylogenetic groups during the spring phytoplankton bloom. *Environ Microbiol* doi: 10.1111/1462-2920.13898

Alonso-Sáez L, Díaz-Pérez L, Morán XAG. (2015) The hidden seasonality of the rare biosphere in coastal marine bacterioplankton. *Environ Microbiol* **17**: 3766–3780.

Calvo-Díaz A, Morán XAG. (2006). Seasonal dynamics of picoplankton in shelf waters of the southern Bay of Biscay. *Aquat Microb Ecol* **42**: 159–174.

Gifford SM, Sharma S, Booth M, Moran MA. (2013) Expression patterns reveal niche diversification in a marine microbial assemblage. *ISME J* **7**: 281-298.

Labonté JM, Swan BK, Poulos B, Luo H, Koren S et al. (2015) Single-cell genomics-based analysis of

virus–host interactions in marine surface bacterioplankton. *ISME J* **9**: 2386-2399
Martinez-Hernandez F, Fornas O, Lluesma Gomez M, Bolduc B, de la Cruz Peña MJ, et al. (2017) Single-cell genomics reveals hidden Cosmopolitan and abundant viruses. *Nature communications* doi: 10.1038/ncomms15892
Mizuno CM, Rodríguez-Valera F, Kimes NE, Ghai R. (2013) Expanding the marine virosphere using metagenomics. *PloS Genetics* **9**: e1003987.
Mizuno CM, Ghai R, Saghai A, López-García P, Rodríguez-Valera F. (2016) Genomes of abundant and widespread viruses from the Deep Ocean. *mBio* **7**: e00805-16
Morán XAG, Alonso-Sáez L, Nogueira E, Ducklow HW, Gonzalez N, et al. (2015) More, smaller bacteria in response to ocean's warming?. *Proc R. Soc. B* **282**: 20150371
Morris RM, Rappé MS, Connon SA, Vergin KL, Siebold WA et al. (2002) SAR11 clade dominates ocean surface bacterioplankton communities. *Nature* **420**: 806-809
Poretsky RS, Gifford SM, Rinta-Kanto J. (2009a) Analyzing Gene Expression from Marine Microbial Communities using Environmental Transcriptomics. *JoVE*. doi: 10.3791/1086.

Table S1. List of phages included in the custom database created for identifying non-cyanobacterial phage transcripts recruiting at least 10 hits in all eight metatranscriptomic datasets combined. The phages were originally detected by BLASTx against the NCBI Refseq database (HTVC019P, HTVC008M, HTVC010P, HTVC011P, HMO-2011) or recently sequenced by single-cell or single-virus genome amplification and metagenomics (i.e., viral fosmids). The SAG phage genomic fragments AAA076E06_contig00007 and AAA160-J18_NODE_19_ID_39 included in the custom database received more than 13 000 BLASTn hits but are not shown in the Table as, after manual inspection, the identified genes were similar to photosynthetic genes and they were likely non-viral hits. Similarly, the alphaproteobacteria prophages PR2-KM22-C70 and PR1-KM20-C273 recruited 611 and 20 hits in the metatranscriptomes but have not been included in the Table. (v)SAG: (viral) Single Amplified Genome.

Acc Num	Phage ID	Putative Host	Blastn hits
NC_020483.1	HTVC019P	SAR11	164
NC_020484.1	HTVC008M	SAR11	63
NC_020481.1	HTVC010P	SAR11	8
NC_021864.1	HMO-2011	SAR116	44
NC_020482.1	HTVC011P	SAR11	34
KY052810.1	vSAG-37-F6	Unknown	177
AP013545.1	S46-C10 (G8)	SAR11	170
KY052816.1	vSAG-37-J6-1	Unknown	133
AP013542.1	S35-C6 (G8)	SAR11	122
AAA164I21_contig00005	AAA164-I21	Verrucomicrobia	101
AAA160P02_contig00022	AAA160-P02	Flavobacteria	100
KT997850.1	GF1-KM16-C1450	Unknown	97
AAA168P09_contig00008	AAA168-P09	SAR86	94
AAA164A08_contig00001	AAA164-A08	Verrucobacteria	73
KT997879.1	GF2-KM19-C266	Unknown	65
AP013386.1	S37-C51 (G15)	SAR11	45
AP013397.1	S25-C46 (G15)	SAR11	45
AP013441.1	S33-C34 (G17)	SAR11	40
AP013551.1	S43-C49 (G15)	SAR11	40
AP013442.1	S36-C18 (G17)	SAR11	37
KT997822.1	GF2-KM20-C144	Unknown	37
AAA160J14_contig00014	AAA160-J14	SAR116	36
KT997833.1	GF0-KM23-C175	Unknown	35
AP013396.1	S44-C25 (G15)	SAR11	34
AP013541.1	S42-C7 (G8)	SAR11	34
KT997844.1	GF1-KM19-C325	Unknown	34
AP013398.1	S30-C38 (G15)	SAR11	33
KY052797.1	vSAG-17-D19-1	Unknown	30
AP013367.1	S31-C11 (G11)	Verrucomicrobia	29
AP013385.1	S39-C44 (G15)	SAR11	29
KT997851.1	GF1-KM16-C1988	Unknown	29
KY052837.1	vSAG-41-A4-1	Unknown	28
AP013359.1	S28-C23 (G1)	Unknown	27
AP013443.1	S23-C7 (G17)	SAR11	27

AP013457.1	S28-C20 (G17)	SAR11	25
AAA164P11_contig00006	AAA164-P11	Verrucomicrobia	23
AP013429.1	S43-C47 (G16)	SAR116	23
AP013558.1	S38-C43 (G15)	SAR11	23
KY052815.1	vSAG-37-I21	Unknown	22
A160J20DRAFT_NODE-unique_1_len_97772.1	AAA160-J20	Thaumarchaeota	21
AAA160D02_contig00016	AAA160-D02	SAR92	20
AP013490.1	S46-C80 (G19)	SAR11	20
KY052843.1	vSAG-41-H4-2	Unknown	20
KY052853.1	vSAG-80-3-I13	Unknown	20
KY052839.1	vSAG-41-D7-1	Unknown	19
AP013430.1	S25-C55 (G16)	SAR116	18
AP013552.1	S39-C49 (G15)	SAR11	18
AP013559.1	S32-C95	SAR11 (Pelagibacter/HIMB114)	18
KY052811.1	vSAG-37-F16	Unknown	18
AAA076E06_contig00001	AAA076-E06	Roseobacter	17
AP013543.1	S30-C28 (G8)	SAR11	17
KY052814.1	vSAG-37-H5-2	Unknown	17
AP013368.1	S25-C42 (G11)	Unkonwn	16
AP013400.1	S30-C37 (G15)	SAR11	16
AP013455.1	S38-C40 (G17)	SAR11	15
KY052809.1	vSAG-37-D17	Unknown	14
KY052840.1	vSAG-41-D7-2	Unknown	14
AAA160C11_contig00006	AAA160-C11	Marinimicrobia	13
AP013554.1	S23-C36 (G15)	SAR11	13
AP013557.1	S41-C64 (G15)	SAR11	13
AAA160D02_contig00008	AAA160-D02	SAR92	12
AP013553.1	S35-C55 (G15)	SAR11	12
AP013556.1	S32-C64 (G15)	SAR11	12
AAA164P11_contig00001	AAA164-P11	Verrucomicrobia	11
AP013406.1	S46-C34 (G15)	SAR11	11
KY052848.1	vSAG-41-I14	Unknown	11
AP013369.1	S43-C17 (G12)	SAR116	10
AP013445.1	S28-C16 (G17)	SAR11	10
AP013485.1	S28-C53 (G19)	SAR11	10
AP013533.1	S38-C34 (G4)	Unknown	10
KT997836.1	CGR0-AD1-C123	SAR11	10
KY052812.1	vSAG-37-G23	Unknown	10

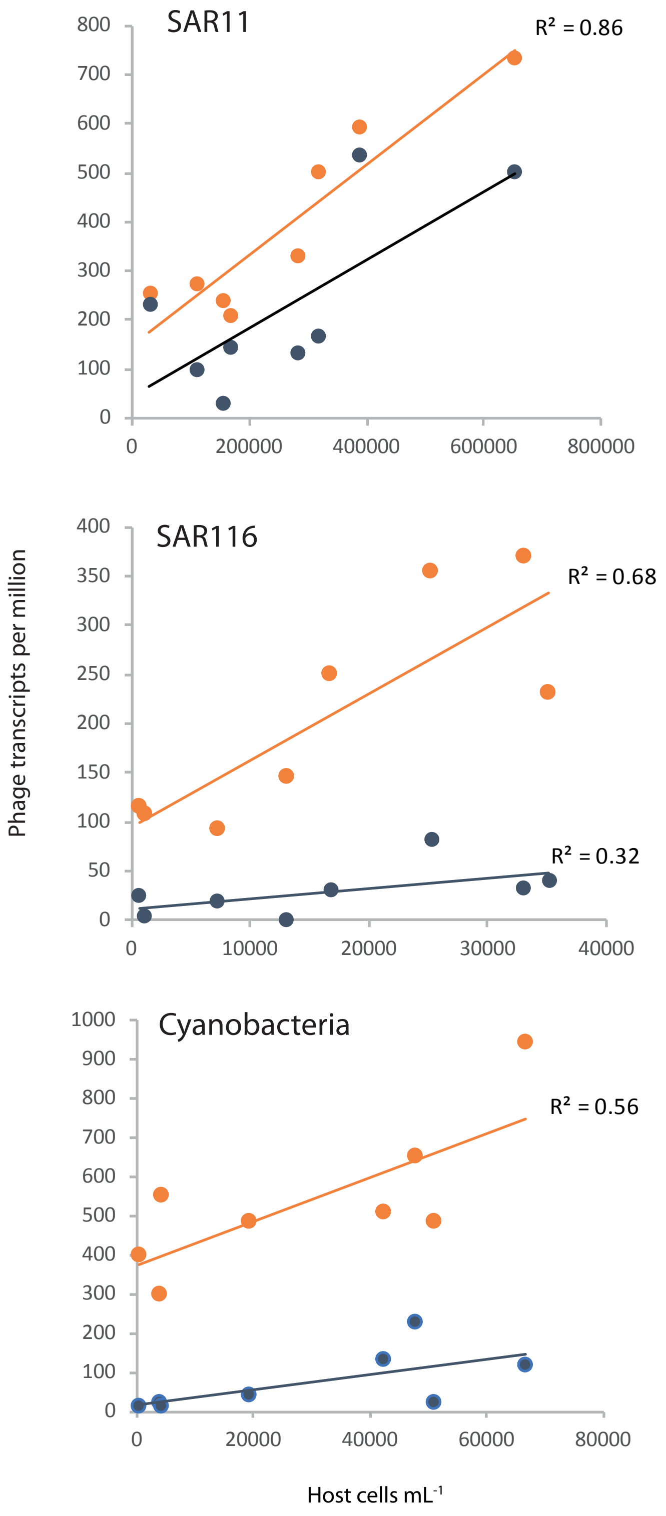

Figure S1. Relationship between the abundance of phage transcripts in the metatranscriptomes and the cell abundance of their respective hosts for SAR11, SAR116 and cyanobacteria. Phage transcripts identified by BLASTx search against Refseq protein database are shown in orange and by BLASTn search against a custom database including phages in Refseq augmented with recently sequenced phages (see Table S1) are shown in blue. In the case of cyanobacteria, results from BLASTx and BLASTn searches were obtained using only the genomes deposited in the Refseq database (see Supplementary text). The abundances of phage transcripts have been normalized by the metatranscriptomic libraries size and expressed as phage transcript per million mRNA reads. The coefficients of determination (R2) for each regression line are shown on the right side for those cases were significant relationships were found (Spearman test, $p \leq 0.05$).

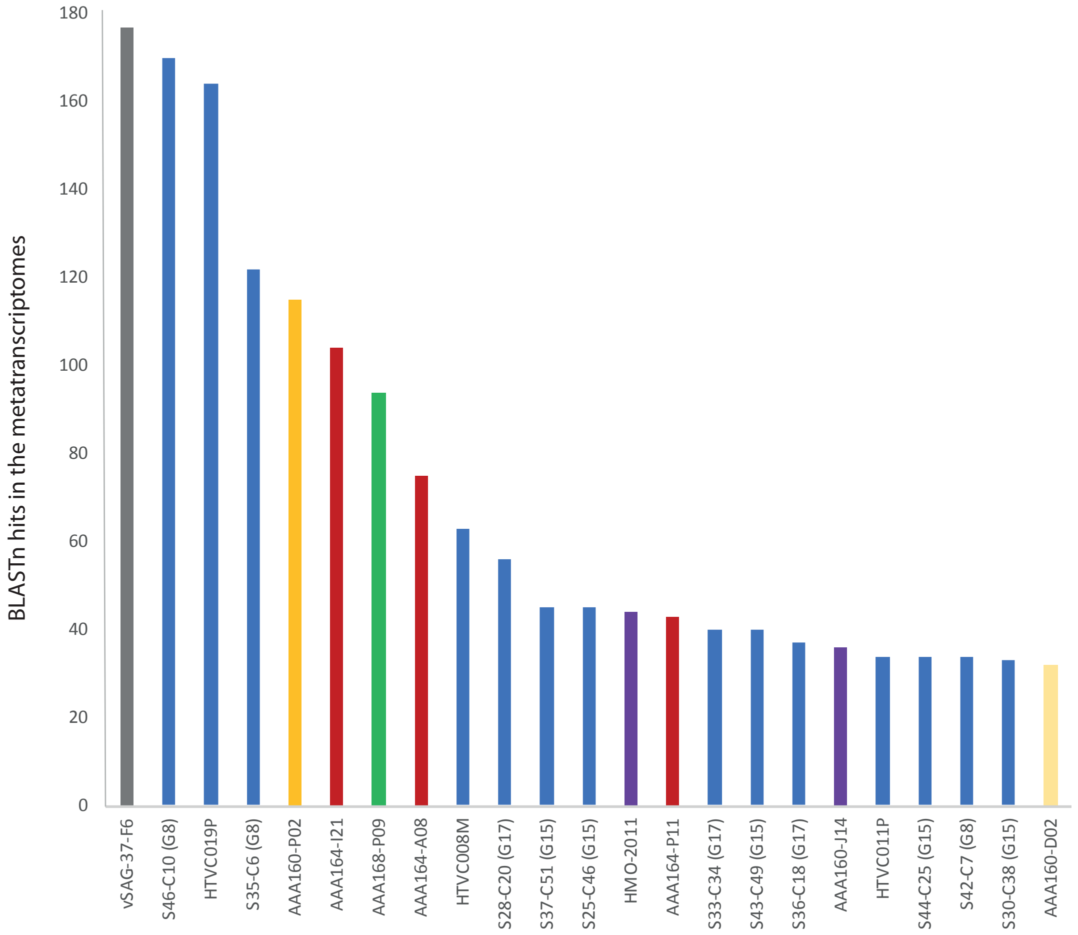

Figure S2. Rank of dominant non-cyanobacterial phages in the metatranscriptomes as detected by nucleotide similarity against a custom database including representative phage genomes (see Table S1). The abundances of phage transcripts are shown as total BLASTn hits in the combined metatranscriptomes (containing 4.2 million mRNA transcripts in total). Phages presumably targeting the SAR11 clade, Flavobacteria, Verrucomicrobia, SAR86, SAR116 and SAR92 clades are shown in blue, orange, red, green, purple and yellow, respectively. Phage vSAG 37-F6, which has no putative hosts identified, has been highlighted in gray.
